# Effects of hydrodynamic cavitation on physicochemical structure and emulsifying properties of tilapia (*Oreochromis niloticus*) myofibrillar protein

**DOI:** 10.3389/fnut.2023.1116100

**Published:** 2023-01-25

**Authors:** Yucheng Hou, Xian’e Ren, Yongchun Huang, Kun Xie, Keyao Wang, Liyang Wang, Fengyan Wei, Feng Yang

**Affiliations:** ^1^Guangxi Key Laboratory of Green Processing of Sugar Resources, Key Laboratory for Processing of Sugar Resources of Guangxi Higher Education Institutes, School of Biological and Chemical Engineering, Guangxi University of Science and Technology, Liuzhou, China; ^2^Guangxi Liuzhou Luosifen Research Center of Engineering Technology, Liuzhou, China

**Keywords:** hydrodynamic cavitation, myofibrillar protein, physicochemical structure, rheology, emulsifying property

## Abstract

The purpose of this research was to explore the different hydrodynamic cavitation (HC) times (0, 5, 10, 15, 20 min; power 550 W, pressure 0.14 MPa) on the emulsifying properties of tilapia myofibrillar protein (TMP). Results of pH, particle size, turbidity, solubility, surface hydrophobicity, and reactive sulfhydryl (SH) group indicated that HC changed the structure of TMP, as confirmed by the findings of intrinsic fluorescence and circular dichroism (CD) spectra. Furthermore, HC increased the emulsifying activity index (EAI) significantly (*P* < 0.05) and changed the emulsifying stability index (ESI), droplet size, and rheology of TMP emulsions. Notably, compared with control group, the 10-min HC significantly decreased particle size and turbidity but increased solubility (*P* < 0.05), resulting in accelerated diffusion of TMP in the emulsion. The prepared TMP emulsion showed the highest ESI (from 71.28 ± 5.50 to 91.73 ± 5.56 min), the smallest droplet size (from 2,754 ± 110 to 2,138 ± 182 nm) and the best rheological properties, as demonstrated by the microstructure photographs. Overall, by showing the effect of HC in improving the emulsifying properties of TMP, the study demonstrated HC as a potential technique for meat protein processing.

## 1. Introduction

Myofibrillar protein (MP), which mainly consists of myosin and actin (1), accounts for 55–65% of the total muscle proteins (2). The emulsifying properties of MP not only affects the quality and performance of emulsified-type meat products (3, 4), such as frankfurter-type sausage (5), but also contributes to the development of nutritive foods with bioactive ingredients as a potential emulsifier (6). However, due to the poor functional properties of natural MP, MP-stabilized emulsions are usually easy to aggregate and flocculate, leading to the difficult in application of MP in the food industry (7). Furthermore, the emulsifying properties of MP are related to protein molecular size and protein conformational characteristics (8). Therefore, modification methods that can cause changes in the conformational characteristics or molecular size of MP may improve the emulsifying properties of MP, thus enhancing the quality of emulsified-type meat products.

In recent years, the physical modification of physicochemical structure of proteins by ultrasonic has attracted researchers’ attention because of its non-toxicity, safety, and environmental friendliness (9). Some authors reported that ultrasonic was used to induce the modification of the functional properties of MP, especially the emulsifying properties (10–12). The underlying mechanism of ultrasonic modification is largely attributed to the cavitation phenomenon during ultrasonic treatment (13). Hydrodynamic cavitation (HC) can produce the same cavitation phenomenon as ultrasonic (14). In HC, cavitation bubbles are generated when the liquid flows through the compression region, and the volume of cavitation bubbles continues to increase as the local pressure continues to decrease (15). However, when the subsequent pressure increases, cavitation bubbles will collapse and explode (16), accompanied by the cavitation effects of instantaneous high temperature and pressure, high-speed micro-jet, strong shock wave, and active free radicals in local areas of the liquid (17). In addition, HC is easier to operate, more energy-efficient and more suitable for scale production than ultrasonic cavitation (18).

Recently, HC treatment, as a novel processing technique, has been reported to enhance the physicochemical and functional properties of proteins. For example, Ren et al. (19) found that HC improved the emulsifying activity index (EAI) and emulsifying stability index (ESI) of soybean protein isolate (SPI), and both increased with the extension of HC treatment time. Yang et al. (17) found that HC improved the EAI, ESI, and adsorbed protein percentage, but decreased the oil droplet size, flocculation index, and interfacial protein concentration, improving the emulsifying properties of SPI. Moreover, HC had some positive effects on the functional properties of soybean glycinin (15) and milk protein concentrate (20). However, the research on the properties of MP modified by HC has not been reported, and the effects of HC on the emulsifying properties of MP are unclear.

Therefore, the objective of this research was to study the effects of different HC times (5, 10, 15, and 20 min) on the physicochemical structure and emulsifying properties of tilapia (*Oreochromis niloticus*) tilapia MP (TMP). The experimental parameters of the pH, particle size, protein solubility, and structural changes in TMP and droplet size, microstructure, and rheological properties of emulsions were tested to elucidate the internal mechanism of HC affecting the emulsifying properties of TMP. The results of this study would provide a theoretical basis and technical support for further study on the functional modification of TMP and production of emulsified-type meat products.

## 2. Materials and methods

### 2.1. Materials

Tilapia, each weighing 750–900 g, were obtained from a local fresh food supermarket (Liuzhou, China). After removing the scales, viscera, skin and bones, the tilapia fish was frozen at −18°C until used. Soybean oil was provided by Kerry Grain and Oil Co., Ltd. (Fangchenggang, China). Bovine serum albumin (BSA) (98%) was purchased from Shanghai yuanye Bio-Technology Co., Ltd. (Shanghai, China). Bromophenol blue (BPB) indicator was purchased from Kermel Chemical Reagent Co., Ltd. (Tianjin, China). 5,5′-Dithiobis-(2-nitrobenzoic acid) (DTNB) (≥98%) was produced by Aladdin Biotechnology Co., Ltd. (Shanghai, China). The other reagents used in the experiment were all analytical grade.

### 2.2. Extraction of TMP

The TMP extraction procedures were adopted from a recent study (21), with slight modifications. Briefly, after small pieces of tilapia fish and a small amount of frozen isolation buffer (25 mmol/L KCl, 3 mmol/L MgCl_2_, 0.1 mol/L NaCl, 4 mmol/L EDTA-2Na, 20 mmol/L phosphate buffer, pH 7.0) were ground in a meat grinder (ZG-L74A, Ningbo Zhao Ji Electric Appliance Co., Ltd., Ningbo, China), the cooling isolation buffer (4°C) was added until the total volume of isolation buffer was five times (w/v) the weight of the tilapia fish. Thereafter, the mixture was homogenized using a homogenizer (Ultra-Turrax T25, IKA, Staufen, Germany) at 9,000 r/min for 2 min (homogenizing for 30 s, then stopping for 30 s). The homogenized mixture was filtered through a layer of gauze to remove some skin and connective tissue, followed by centrifugation using a high-speed refrigerated centrifuge (JXN-26, Beckman Coulter Inc., Brea, CA, USA) at 3,220 × *g* at 4°C for 15 min. Then, the precipitate was homogenized and centrifuged twice under the same conditions with cooling five times (w/v) of isolation buffer (4°C) and four times (w/v) of 0.1 mol/L NaCl solution (4°C), respectively. The precipitate was then added to four times (w/v) of cooling phosphate buffer solution (20 mmol/L, pH 7.0, 4°C) and homogenized at 9,000 r/min for 2 min (homogenizing for 30 s, then stopping for 30 s). Thereafter, the mixture was filtered through three layers of gauze followed by centrifugation at 3,220 × *g* at 4°C for 15 min to obtain the precipitate. The protein concentration of TMP suspension was determined by the biuret method (22), using an ultraviolet-visible spectrophotometer (UV-5100, Shenzhen Ecorui Instrument Equipment Co. Ltd., Shenzhen, China) with BSA as the standard.

### 2.3. Treatment of TMP by HC

**FIGURE 1:**
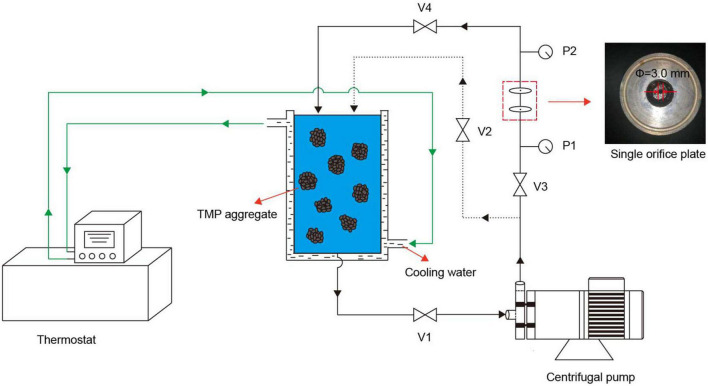
Schematic diagram of the hydrodynamic cavitation (HC) experimental setup. The HC device consists of a centrifugal pump (power 550 W), a storage tank, a thermostat, a single orifice plate (orifice diameter 3 mm, thickness 20 mm), two pressure gauges (P1 and P2), and four valves (V1, V2, V3, and V4).

The HC treatment was carried out as described by previous studies with slight modifications (15, 19). As shown in Figure 1, V1 was used to adjust the flow of TMP suspension, V2 and V3 were used to adjust the upstream inlet pressure, which was measured by P1, and V4 was used to adjust the downstream recovery pressure. The TMP was dissolved in 0.6 mol/L NaCl buffer (0.6 mol/L NaCl, 20 mmol/L NaH_2_PO_4_/Na_2_HPO_4_, pH 6.5) and homogenized at 9,000 r/min for 30 s to prepare 10 mg/ml TMP suspension. After pouring 900 ml of TMP suspension into a storage tank and turning on the cooling water, the HC treatment of TMP suspension was performed at a pressure of 0.14 MPa and a power of 550 W, using a single orifice plate (thickness 20 mm, orifice diameter 3 mm). After HC treatment for 0, 5, 10, 15, and 20 min, the TMP samples were stored at 4°C for further analysis.

### 2.4. Physicochemical properties

#### 2.4.1. pH

The pH of TMP samples was measured as described by Wang et al. (21), using a pH meter (PHS-3E, INASE Scientific Instrument Co., Ltd., Shanghai, China).

#### 2.4.2. Particle size

The TMP particle size was analyzed using the method of Chen et al. (6). The average particle sizes of diluted TMP (1 mg/ml) samples were determined at 25°C, using a Zetasizer (Nano-ZS90, Malvern Instrument Co. Ltd., Malvern, UK). The refractive index of particles, refractive index of dispersant, equilibrium time, and absorption parameter were 1.46, 1.33, 2 min, and 0.01, respectively.

#### 2.4.3. Turbidity

A recent study was followed for turbidity measurement with slight modifications (23). Briefly, the absorbance of TMP was performed at 660 nm after the diluted TMP samples (1 mg/ml) were placed at room temperature for 30 min, using the UV-5100 spectrophotometer. A_660_ was used to indicate the turbidity of the TMP samples.

#### 2.4.4. Protein solubility

The protein solubility of TMP samples was measured according to a published method (24), using BSA as the standard protein, with slight modifications. Briefly, 8 ml of the TMP samples (1 mg/ml) were taken and centrifuged at 10,000 × *g* for 10 min. Then, 1 ml of supernatant and 1 ml of TMP samples without centrifugation were added to 4 ml of biuret reagent, respectively. After mixing well, the absorbance was measured at 540 nm after reaction for 30 min in a dark chamber at room temperature. The protein solubility was expressed as follows:


$$\text{S}\left(\%\right)=\frac{{\text{C}}_{\text{a}}}{{\text{C}}_{b}}\times 100$$

Where S, C_b_, and C_a_ represent the protein solubility of TMP samples (%), total protein content before centrifugation and protein content of the supernatant, respectively.

### 2.5. Secondary and tertiary structures

#### 2.5.1. Surface hydrophobicity (H_0_)

The H_0_ was analyzed as described by Jia et al. (25). Briefly, 1 ml of TMP sample (5 mg/ml) was added to 200 μL of BPB solution (1 mg/ml) and mixed evenly, using a mixture of 200 μL of BPB solution (1 mg/ml) and 1 ml of 0.6 mol/L NaCl buffer as control. After standing at room temperature for 2 h and centrifuging at 6,000 × *g* for 15 min, 0.5 ml of supernatant was diluted into 4.5 ml of 0.6 mol/L NaCl buffer, and the absorbance of the diluted supernatant was measured at 595 nm using the UV-5100 spectrophotometer. The amount of BPB bound to TMP was used to represent the surface hydrophobicity of TMP, which was calculated as follows:


$$H_{0}\,\left(\mu \,g\right)=200\,\mu \,g\times \frac{A_{1}-A_{2}}{A_{1}}$$

Where A_1_ and A_2_ represent the absorbance value of control and TMP samples, respectively.

#### 2.5.2. Reactive sulfhydryl (SH) group

The reactive sulfhydryl (SH) group of TMP samples was examined according to a previously published method (26). Briefly, after 50 μL of 10 mmol/L DTNB solution (phosphate buffer, pH 8.0) was mixed with 4 ml of TMP sample (1 mg/ml) and then incubated at 25°C for 20 min, the absorbance of the mixture was recorded at 412 nm using the UV-5100 spectrophotometer. The reactive SH group content was expressed as follows:


$$S\,H\,\left(\mu \,m\,o\,l/100\,\,m\,g\right)\,=\frac{A_{412}\times D}{E_{M}\times C}\times 100,000$$

Where A_412_, D, E_M_, and C represent the absorbance of the mixture at 412 nm, dilution factor, molar extinction coefficient (13,600 L⋅mol^–1^⋅cm^–1^) and TMP concentration (mg/ml), respectively.

#### 2.5.3. Intrinsic fluorescence spectroscopy

The intrinsic fluorescence spectra were analyzed using a Cary Eclipse fluorescence spectrophotometer (G9800A, Agilent Technologies Ltd., Santa Clara, CA, USA) with slight modifications (11). Briefly, after the TMP samples were dissolved in 0.6 mol/L NaCl buffer and adjusted to 0.2 mg/ml, the intrinsic fluorescence spectra of TMP samples were recorded at excitation wavelength, emission wavelength, excitation slit width, and emission slit width of 280, 300–400, 5, and 5 nm, respectively.

#### 2.5.4. Circular dichroism (CD) spectroscopy

The circular dichroism (CD) spectra were measured using a CD spectropolarimeter (Chirascan, Applied Photophysics Ltd., Surrey, UK) with slight modifications (27). Briefly, after the TMP samples were diluted to 0.067 mg/ml and then injected into a quartz cuvette, the CD spectra were scanned in the far-UV range (200–260 nm) at room temperature. The results were described by the average of three scans, and the circle two data were expressed by the average molar ellipticity (θ) in deg⋅cm^2^/dmol. The scan rate, response time, bandwidth, and sensitivity were 100 nm/min, 0.5 s, 1.0 nm, and 20 mdeg, respectively. The proportions of four secondary structures were analyzed by CDNN software.

### 2.6. Emulsifying properties

#### 2.6.1. Emulsion preparation

The preparation of O/W emulsion followed a recent report (6), with slight modifications. Briefly, 28 ml of TMP samples (10 mg/ml) were added to 7 ml of soybean oil, followed by homogenization at 8,000 r/min for 1 min.

#### 2.6.2. EAI and ESI

The EAI and ESI of TMP emulsions were examined as reported (11). Briefly, immediately (0 min) and 10 min later after homogenization, 50 μL of TMP emulsion of the bottom was diluted to 5 ml of 0.1% sodium dodecyl sulfate (SDS) (w/v) solution. After mixing well, the absorbance of the mixture was measured at 500 nm by the UV-5100 spectrophotometer, using SDS solution as blank. EAI and ESI were calculated as follows:


$$E\,A\,I\,\left(m^{2}/g\right)\,=\frac{2\times 2.303\times A_{0}\times N}{C\times \theta \times 10000}\,$$

$$E\,S\,I\,\left(m\,i\,n\right)\,=\frac{A_{0}}{A_{0}-A_{10}}\times 10$$

Where N, θ, and C represent the dilution factor (100), volume fraction of oil in emulsion (0.2) and initial concentration of TMP (g/ml), respectively, and A_0_ and A_10_ represent the absorbance at 500 nm after 0 and 10 min, respectively.

#### 2.6.3. Droplet size of the emulsions

The droplet size measurement was performed using the Nano-ZS90 Zetasizer according to the published method (11). Briefly, about 1 ml of fresh emulsion diluted 500-fold with 0.6 mol/L NaCl buffer was loaded into a quartz cuvette to measure droplet size.

#### 2.6.4. Microstructure of the emulsions

The microstructure of TMP emulsions was observed using an optical microscope (DM-2000 LED, Leica Microsystems Co. Ltd., Wetzlar, Germany) (6). Briefly, 10 μL of the homogenized emulsion was dropped in the center of the slide and slowly covered with a cover glass to ensure no bubbles formed. The image was obtained by a 20× objective lens.

#### 2.6.5. Dynamic rheological measurements of the emulsions

The rheology of TMP emulsions was examined using a rotational rheometer (MCR72, Antompa, Graz, Austria) with slight modifications (28). Briefly, using a 50 mm plate test, 3 ml of TMP emulsion was evenly coated on the test platform, and a layer of silicone oil was applied around TMP emulsion to prevent evaporation of TMP emulsion during the heating process. The frequency, strain, initial temperature, heating rate, termination temperature, and crack spacing were 0.1 Hz, 1%, 20°C, 1°C/min, 80°C, 1.0 mm, respectively. Storage modulus (G′) and loss modulus (G″) were analyzed as test indexes.

### 2.7. Statistical analysis

Each trial was conducted in triplicate independently and the data were presented as the mean ± standard deviation (SD). One-way analysis of variance (ANOVA) and Duncan’s test were analyzed using SPSS statistical software (Version 26.0, IBM Corp., Armonk, NY, USA) to determine statistical differences (*P* < 0.05) among different groups.

## 3. Results and discussion

### 3.1. pH

The changes in the pH of TMP suspension as affected by HC treatment are shown in Table 1. Compared with control group (0 min), HC increased the pH of TMP suspension significantly (*P* < 0.05) by approximately 0.06 units (Table 1), indicating that HC induced a pH transition to alkaline, which was beneficial to the solubilization of TMP (Table 1) (21). However, with the extension of HC treatment time, pH only decreased slightly, indicating that pH was not the main factor in solubility reduction, which might be due to the thermal effect generated by long HC treatment time (17), resulting in TMP aggregation. The increase of pH might be because the cavitation effect produced by HC leaded to the denaturation of TMP and the generation of free radicals, which reacted with protein side chains to reduce the acidic groups of protein. Similar speculations were reported in the results that ultrasonic treatment increased the pH of MP suspensions (10). In addition, a recent study reported that HC changed the pH of SPI suspension, and inferred that HC affected the exposure of acidic or basic amino acid residues (19). Therefore, the amount of acidic and basic amino acid residues exposed might be another important factor affecting the pH of the TMP suspension. However, there was no significant difference in pH between HC treatment groups (*P* > 0.05), which might be due to the similar amount of acidic and basic amino acid residues exposed. Nevertheless, Ren et al. (19) found that neither HC nor ultrasound treatment significantly changed the pH of SPI suspensions. The difference in results might be due to differences in the type of protein and cavitation.

**TABLE 1:** Effect of hydrodynamic cavitation (HC) on the pH, average particle size, turbidity, and protein solubility of tilapia myofibrillar protein (TMP).

Time (min)	pH	Average particle size (nm)	Turbidity	Protein solubility (%)
0	6.33 ± 0.02^b^	1,026.2 ± 25.9^b^	0.111 ± 0.001^b^	57.65 ± 1.08^c^
5	6.39 ± 0.01^a^	719.3 ± 41.1^c^	0.093 ± 0.001^d^	63.07 ± 1.37^ab^
10	6.39 ± 0.01^a^	681.6 ± 13.6^c^	0.089 ± 0.001^d^	64.90 ± 0.34^a^
15	6.38 ± 0.01^a^	967.3 ± 27.8^b^	0.105 ± 0.004^c^	59.50 ± 0.41^bc^
20	6.38 ± 0.01^a^	2,095.3 ± 61.6^a^	0.227 ± 0.003^a^	29.44 ± 5.10^d^

Data are presented as mean ± standard deviation (SD) (*n* = 3). Different lowercase letters indicate that TMP had significant differences at different HC times (*P* < 0.05).

### 3.2. Particle size, turbidity, and protein solubility

The average particle size of TMP decreased with the increase of HC treatment time within 0–10 min, as shown in Table 1. Furthermore, compared with 0, 15, and 20 min, HC for 5 min (from 1,026.2 ± 25.9 to 719.3 ± 41.1 nm) and 10 min (from 1,026.2 ± 25.9 to 681.6 ± 13.6 nm) decreased the average particle size of TMP significantly (*P* < 0.05), while no significant differences were observed between the two HC treatments (*P* > 0.05). This decrease might be attributed to cavitation effects during HC treatment, such as turbulence, high shear, and shock waves, which destroyed the non-covalent bonds between TMP molecules, such as hydrogen bonds and electrostatic interactions, causing the dissociation of TMP. Similar results have been reported that HC treatment decreased the particle size of SPI (17, 19) and soybean glycinin (15). However, the average particle size of TMP increased significantly (*P* < 0.05) with the extension of HC treatment time within 10–20 min. The increase in particle size of TMP might be due to high temperature and excessive free radicals caused by excessive HC, which leaded to the acceleration of movement and the increase of collision opportunities of TMP molecules, promoting aggregation of TMP. Similar results were observed for the effects of ultrasonic treatment on the particle size of MP (7, 12, 29). These results showed that appropriate HC could decrease effectively the formation of large aggregates, which might benefit the application of TMP in emulsification.

The turbidity of TMP as affected by HC is shown in Table 1. In general, higher turbidity corresponds to denser protein aggregation (6). HC for 10 min had lower turbidity (0.089 ± 0.001) compared to the other groups, which could be explained by a decrease in particle size, which resulted in less light scattering (30). However, with the further increase in HC treatment time (10–20 min), the turbidity of TMP increased significantly (*P* < 0.05), which might be due to the de-folding and reaggregation of protein molecules affecting light scattering, leading to an increase in turbidity (31). Additionally, excessive HC could decompose water and generate active free radicals (15), promoting intramolecular or intermolecular protein cross-linking, resulting in increased turbidity.

The solubility of TMP increased with the increase in HC treatment time (0–10 min), as shown in Table 1. Compared with 0, 15, and 20 min, HC for 10 min significantly increased the solubility of TMP (*P* < 0.05), which might be due to cavitation effects that dissociated TMP to form smaller aggregates with larger surface area and enhanced protein-water interaction, resulting in better solubility (32). However, as HC treatment time (10–20 min) continued to increase, protein solubility decreased significantly (*P* < 0.05), which might be caused by TMP aggregation (31). Therefore, appropriate HC could dissociate the TMP aggregate, resulting in increased solubility. In addition, the changes in particle size, turbidity, and solubility were consistent, confirming the significant influence of HC on the dissociation of TMP aggregates.

### 3.3. Tertiary structures

#### 3.3.1. H_0_

**FIGURE 2:**
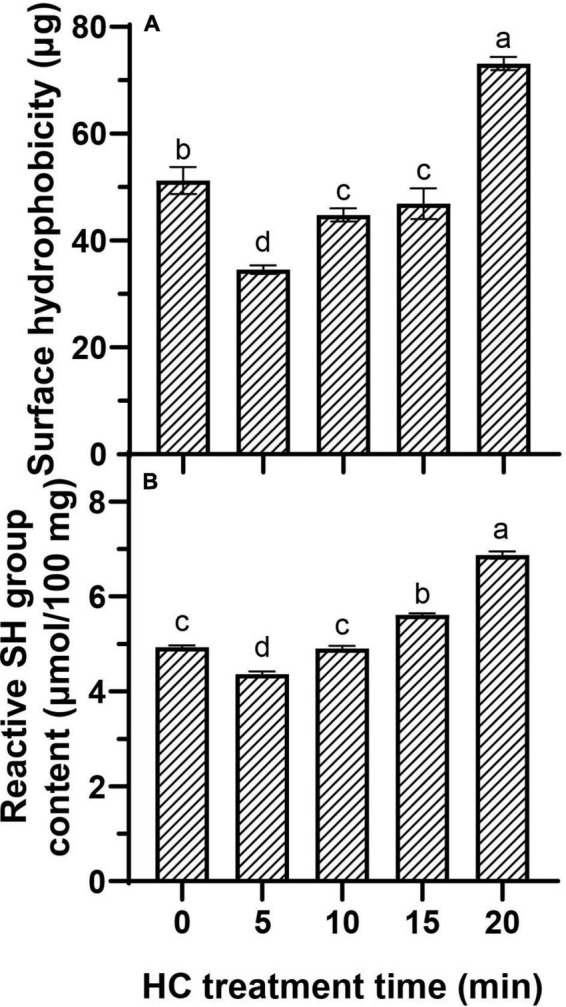
Effect of hydrodynamic cavitation (HC) on the surface hydrophobicity (H_0_) **(A)** and reactive sulfhydryl (SH) group **(B)** of tilapia myofibrillar protein (TMP). Different lowercase letters indicate that TMP had significant differences at different HC times (*P* < 0.05).

The H_0_ represents the exposure of hydrophobic groups in protein molecules (29). This work is to determine the H_0_ by analyzing the binding of hydrophobic amino acids of proteins to BPB. Compared with control group, HC for 5 min significantly decreased the binding amount of BPB (*P* < 0.05; Figure 2A). The decrease in H_0_ was based on an increase in protein solubility (Table 1) (33), since the hydrophobic amino acids of TMP might be distributed inside the molecule by HC, leading to enhancing protein-water interactions and exposing less hydrophobic groups (34). With the further increase of HC treatment time (5–20 min), the H_0_ of TMP increased significantly (*P* < 0.05), and reached the highest value (73.11 ± 1.23 μg) after HC treatment for 20 min. This was because the cavitation effect unfurled TMP structure and exposed hydrophobic groups, resulting in increased H_0_. Similar to other physical modified MP studies (35–37). In addition, the temperature effect of HC in the local region of the suspension may be another important factor that causes the conformational change of TMP. Similarly, Pan et al. (30) suggested that the temperature effect of ultrasonic might alter the tertiary structure of MP due to the susceptibility of fish MP to thermal denaturation. These results suggested that HC changed the H_0_ due to its influence on protein-water and protein intermolecular interactions and the exposure of hydrophobic groups.

#### 3.3.2. Reactive SH group

The reactive SH groups refer to the SH groups located on the surface of the protein network, which can reflect MP tertiary structure conformational changes (7). Compared with control group, the reactive SH content was decreased significantly after HC treatment for 5 min (*P* < 0.05; Figure 2B), indicating that the structure of TMP and disulfide bond formation were changed, possibly because the reactive SH on TMP surface was converted to disulfide bonds by the HC-induced cavitation effects. Similarly, Yang et al. (17) reported that HC could lead to the conversion of free SH into disulfide bonds in SPI. However, compared with the 5 min, the reactive SH content increased significantly with the increase of HC treatment time (*P* < 0.05), indicating that HC accelerated the expansion and structural changes of protein molecules, leading to exposing more SH group hidden inside the protein molecules (19).

### 3.4. Emulsifying properties

#### 3.4.1. EAI and ESI

**FIGURE 3:**
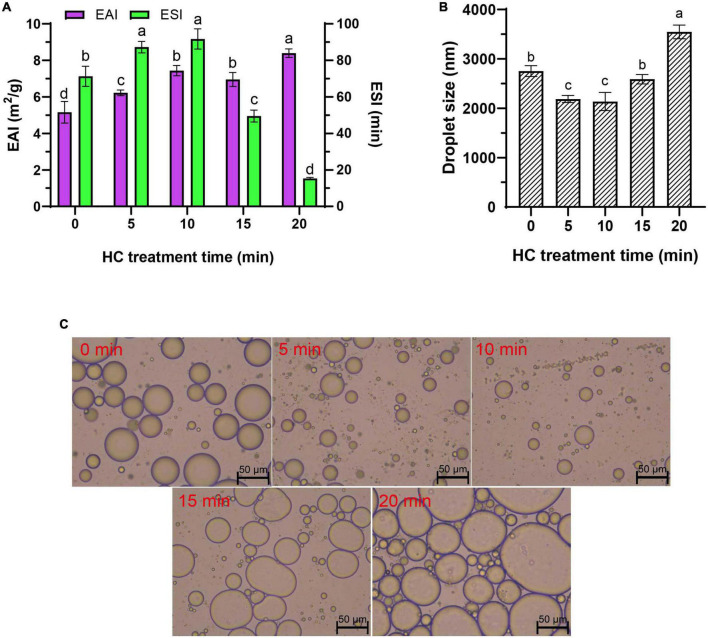
Effect of hydrodynamic cavitation (HC) on the emulsifying activity index (EAI) and emulsifying stability index (ESI) **(A)**, droplet size **(B)**, and microstructure **(C)** of tilapia myofibrillar protein (TMP) emulsions. In panels **(A,B)** different lowercase letters indicate that TMP emulsions had significant differences at different HC times (*P* < 0.05).

EAI and ESI represent the capacity of a protein to be adsorbed by the interface between the water phase and oil sphere during the formation of protein emulsion and the ability of a protein to maintain at the oil-water interface of emulsion after storage period, respectively (6, 24). Compared with control group (0 min), all HC treatments significantly increased the EAI of TMP emulsion (*P* < 0.05), and EAI was increased significantly with the increase of HC treatment time (except 10 and 15 min) (*P* < 0.05; Figure 3A). Different from EAI, ESI showed a trend of increasing first and then decreasing, that is, increasing gradually within 0–10 min of HC treatment and decreasing gradually with further treatment (10–20 min). The increase in EAI and ESI of TMP might be due to the cavitation effect generated by HC that decreases the particle size of TMP (Table 1), making more TMP easily adsorbed on the oil-water interface, resulting in the improvement of emulsifying capacity (17). Furthermore, lower protein turbidity and higher solubility (Table 1) accelerated the diffusion of TMP in the emulsion (6, 23). Interestingly, excessive HC treatment (20 min) increased particle size and turbidity and decreased solubility (Table 1) compared with the other groups, leading to a decrease in ESI but not in EAI as expected. This phenomenon might be attributed to an increase in H_0_ (Figure 2A), which led to the enhancement of the interaction between adjacent molecules at the oil-water interface (7). Some other studies have reported that MP (10, 12) and SPI (17, 19) showed a good positive correlation between EAI and H_0_, and increasing H_0_ was conducive to improve EAI of proteins. This study showed that HC for 10 min could improve the EAI and ESI of TMP significantly.

#### 3.4.2. Droplet size

Droplet size determines the rheology and stability of emulsion, and generally smaller droplet size is associated with higher emulsifying capacity (23, 38). The droplet size of TMP emulsion decreased significantly from 2,754 ± 110 nm (0 min) to 2,138 ± 182 nm (10 min) (*P* < 0.05) with the increase in HC treatment time (0–10 min), but no significant difference was observed between 5 min and 10 min treatments (*P* > 0.05; Figure 3B). However, with the continuous extension of HC treatment time (10–20 min), the droplet size of TMP emulsion increased significantly (*P* < 0.05), and this opposite trend was attributed to the overtreatment of the protein (7). These results indicated that appropriate HC could be conducive to decrease the droplet size of TMP emulsion and improve its emulsifying properties.

#### 3.4.3. Microstructure of the emulsions

The microstructure of TMP emulsions as affected by HC was enlarged 200 times under an optical microscope, as shown in Figure 3C. The soybean oil droplets in the control group (0 min) were relatively large and unevenly distributed, which was attributed to the high interfacial tension between water and soybean oil (6). Compared with the untreated group, HC for 10 min had fewer large oil droplets, suggesting that HC induced TMP expansion and conformational changes, thereby decreasing the interfacial tension between water and soybean oil. Similar results have been reported in ultrasonic modification of MP (6). However, it was observed that the oil droplets size in the fresh emulsion increased, and the distribution was more uneven with the further extension of HC treatment time (10–20 min). The results of optical microscopic images could intuitively show the oil droplet size, corresponding to the previous results of the droplet size (Figure 3B). These results showed that compared with control group, HC for 10 min decreased the oil droplet size and improved the uniformity of emulsion.

#### 3.4.4. Rheological properties of the emulsions

**FIGURE 4:**
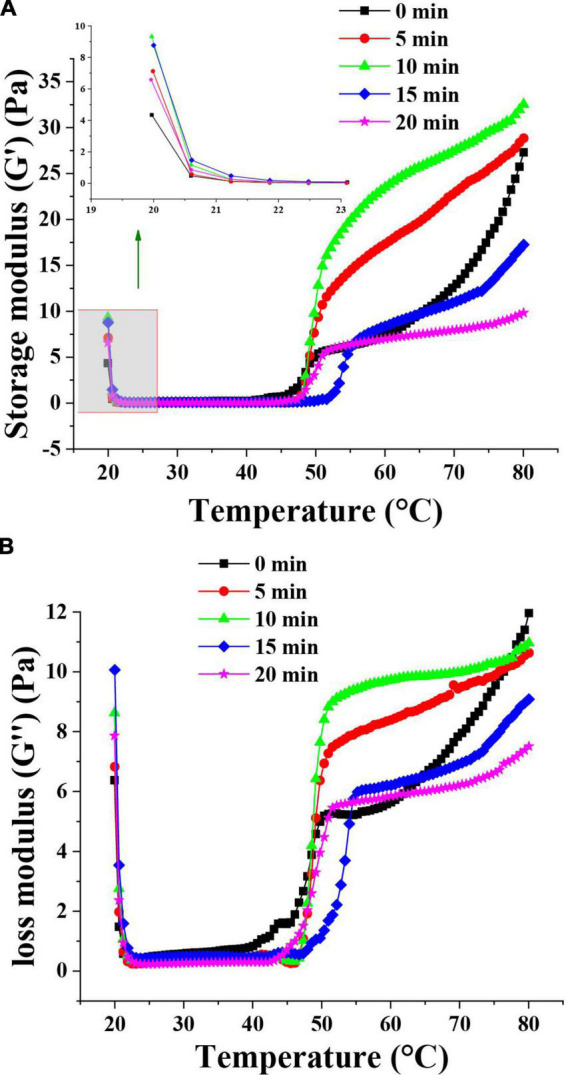
Effect of hydrodynamic cavitation (HC) on the storage modulus (G′) **(A)** and loss modulus (G″) **(B)** of tilapia myofibrillar protein (TMP) emulsions. The inserted figure is the enlarged part of the control and HC treatment groups.

The rheological behavior of emulsions prepared with MP was analyzed to understand the viscoelastic properties of MP emulsions and the functionality of muscle protein in meat processing (39). The rheological properties of the TMP emulsions as affected by HC are shown in Figure 4. The G′ of TMP emulsion showed a decrease at the initial stage of heating (20–22°C), which might be attributed to the formation of weak and preliminary interactions of the protein aggregates prior to heating and destruction after initial heating (11). Compared with other groups, HC for 10 min had the highest G′ value at the initial stage and scanning end, indicating that HC for 10 min had the most robust gel structure of TMP emulsion (28). In addition, after HC treatment for 10 min, the particle size of TMP was decreased and an emulsion with more uniform and smaller oil droplets was formed. HC for 10 min decreased the particle size of TMP and formed smaller and more uniform oil droplets in the emulsion. Li et al. (11) showed that smaller oil droplets could fill effectively and evenly into the network of MP gel during heating, resulting in better elasticity of MP emulsion gel. Therefore, HC for 10 min improved the elastic properties of TMP emulsion and prepared emulsion with stronger rheological properties. The changes in G″ of TMP emulsions were similar to that of G′. The G″ value of the TMP emulsion treated by HC for 10 min during about 50∼70°C heating was higher than that of the control (0 min). For all emulsions, the G″ values were substantially lower than that of G′ values indicating the formation of a gel-like behavior (39). The values of G′ and G″ increased with the increase of HC treatment time, indicating that HC promoted the formation of firm and dense gel structure of proteins. However, with the further extension of HC treatment time, the viscoelastic properties of the emulsion decreased, possibly due to the formation of uneven and rough gel structure. Rheological measurements showed that appropriate HC (10 min) improved the rheological properties of TMP-based emulsion as well as the structure of TMP.

### 3.5. Schematic model

**FIGURE 5:**
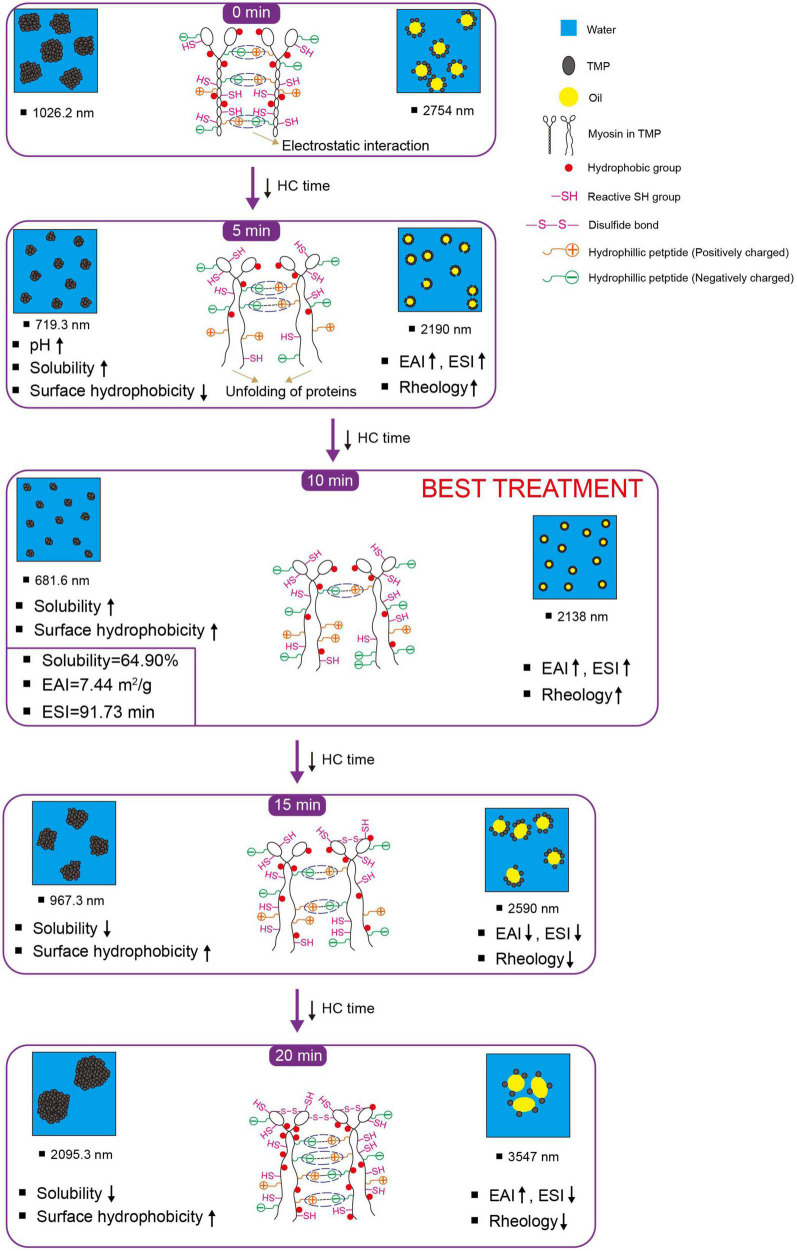
Schematic model illustrating the effects of hydrodynamic cavitation (HC) treatment on physicochemical structure and emulsifying properties of tilapia myofibrillar protein (TMP). ESI, emulsifying stability index; EAI, emulsifying activity index; reactive SH group, reactive sulfhydryl group. The chemical structure is not represented to actual scale, just to illustrate (For color references in this illustration, please refer to the online version of this article).

Based on the results of physicochemical properties (sections “3.1. pH,” and “3.2. Particle size, turbidity, and protein solubility”), tertiary structures (section “3.3. Tertiary structures”), and emulsifying properties (section “3.4. Emulsifying properties”), a schematic model (Figure 5) was proposed for the presentation of the modifications of HC-induced TMP. HC changed the particle size, turbidity, and solubility of TMP (Table 1) due to cavitation effects such as high shear, shock wave, and turbulence generated during HC treatment that affected covalent bonds such as disulfide bonds and non-covalent bonds such as electrostatic interactions between protein molecules (15, 17). Additionally, HC changed the tertiary structure of TMP (Figure 2) and partially expanded the TMP. These results led to changes in the interaction between protein and oil phases (7, 11, 40), thus affecting the emulsifying (Figure 3) and rheological (Figure 4) properties of TMP emulsions. Notably, HC for 10 min decreased the average particle size and turbidity and increased the solubility of TMP significantly (*P* < 0.05), which was conducive to the diffusion of TMP in the emulsion (6, 23). In addition, HC for 10 min increased the EAI and ESI significantly (*P* < 0.05), decreased the droplet size of TMP emulsion significantly (*P* < 0.05), and made its distribution more uniform, resulting in improved emulsifying and rheological properties of TMP emulsion. In summary, TMP treated by HC for 10 min had the capacity to form more uniform and smaller emulsion droplets. Interestingly, the decrease in emulsifying properties of TMP treated by HC for 20 min might be due to the excessive oxidation of TMP resulting in more reactive SH conversion to disulfide bonds (17), as well as the increase in particle size and decrease in solubility (Table 1). Therefore, this study showed that HC for 10 min enhanced the stability of emulsion stabilized by TMP and emulsifying properties of TMP.

### 3.6. Validation of the schematic model

#### 3.6.1. Intrinsic fluorescence spectra

**FIGURE 6:**
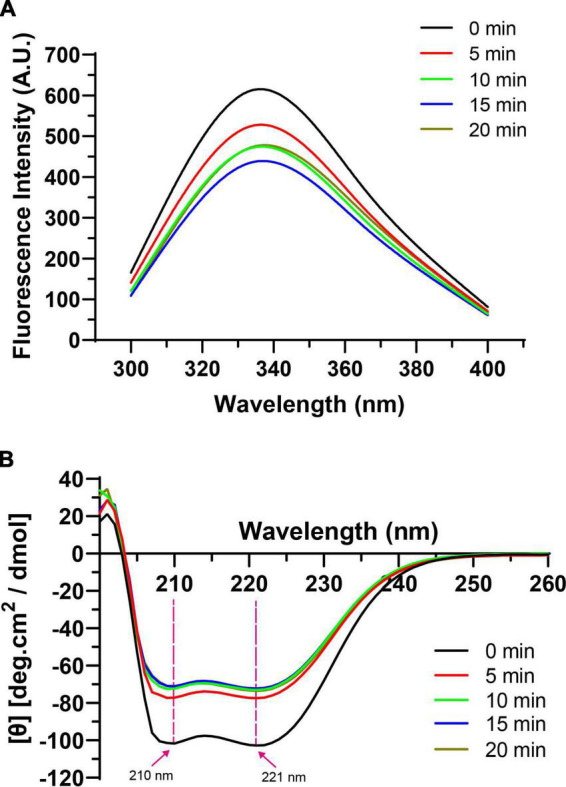
Effect of hydrodynamic cavitation (HC) on intrinsic fluorescence spectra **(A)** and circular dichroism (CD) spectra **(B)** of tilapia myofibrillar protein (TMP).

The tertiary and secondary structure changes of the HC-induced TMP were measured by intrinsic fluorescence spectra and CD spectra, which reflected the changes in the physicochemical structure and emulsifying properties of TMP to verify the proposed schematic model. In general, intrinsic fluorescence spectroscopy is used to assess the local environment of certain chromophores, especially tryptophan residues, and can monitor tertiary structural changes in proteins (7). The fluorescence intensity of all treatment groups was lower than that of the control group (Figure 6A), suggesting that HC destroyed hydrophobic interactions, allowing the structure of TMP molecules to unfold, which caused the movement of tryptophan residues to more polar environments. Similar results have been reported in the ultrasonic treatment of MP (30, 31). For example, Jiang et al. (31) speculated that cavitation effects generated by ultrasound exposed the tryptophan group to the solvent, leading to a decrease in fluorescence intensity of MP. Pan et al. (30) pointed out that ultrasound disrupted hydrophobic interactions between MP molecules and moved tryptophan residues to a more polar environment. Interestingly, the fluorescence intensity of TMP decreased when HC treatment time increased from 0 to 15 min but increased after 20 min. This might be due to protein aggregation (Table 1), increased H_0_ (Figure 2A), and specific modification of tryptophan residues in TMP, which combinedly resulted in less exposure of tryptophan residues to water (12).

In addition, in terms of the maximum fluorescence emission wavelength (λ_max_), the λ_max_ of the control group (334 nm) was higher than 330 nm, indicating that tryptophan was in a polar environment (41). The larger the redshift of λ_max_, the larger the conformational change of the protein. With the extension of HC treatment time, λ_max_ of TMP redshifted from 334 to 338 nm, which might be due to the exposure of tryptophan and the microenvironment becoming more polar, causing the tertiary conformation of TMP to become looser (Figure 5) (12).

#### 3.6.2. CD spectra

The CD spectra of TMP as affected by HC are shown in Figure 6B. In general, the α-helical structure is formed mainly by the hydrogen bond between the carbonyl oxygen (C=O) and amino hydrogen (NH^–^) groups in the polypeptide chain (42). Two negative peaks of α-helical structures were detected at 210 and 221 nm, and HC decreased the intensity of these two peaks, indicating that HC destroyed the intramolecular hydrogen bond of TMP, thus promoting the unfolding of the protein (43). At the same time, Table 2 also shows that HC significantly decreased the α-helix content (*P* < 0.05). Additionally, compared with control group, the β-sheet, β-turn, and random coil contents of all HC treatment groups were increased significantly (*P* < 0.05), but no significant differences were observed between HC treatment groups (*P* > 0.05). The transformation of secondary structure of TMP might be caused by the hydrogen bond change induced by HC. Similar conclusions have been reported in using other physical modification techniques to change the secondary structure of MP, such as ultrasound (44), high-pressure homogenization (23). Moreover, Yang et al. (17) reported that swirling cavitation increased β-sheet content and decreased α-helix content, which might improve the emulsifying properties of SPI. Consistent with the experimental results, HC increased the β-sheet content and decreased the α-helix content of TMP significantly (*P* < 0.05) compared with control group, which might be another important reason for the enhancement of emulsifying properties of TMP (Figures 3, 4). However, the decrease in emulsifying properties after HC for 15 and 20 min might be attributed to the increase in particle size and turbidity as well as the decrease in solubility (Table 1).

**TABLE 2:** Effect of hydrodynamic cavitation (HC) on the secondary structure contents (%) of tilapia myofibrillar protein (TMP).

Time (min)	Secondary structure of TMP			
	**α-Helix**	**β-Sheet**	**β-Turn**	**Random coil**
0	53.4 ± 0.7^a^	9.9 ± 0.2^b^	14.0 ± 0.2^b^	22.7 ± 0.3^b^
5	39.3 ± 2.6^b^	14.8 ± 1.2^a^	15.9 ± 0.4^a^	30.0 ± 1.4^a^
10	37.9 ± 2.1^b^	15.3 ± 0.9^a^	16.1 ± 0.3^a^	30.7 ± 1.3^a^
15	38.1 ± 0.4^b^	15.2 ± 0.2^a^	16.1 ± 0.2^a^	30.6 ± 0.4^a^
20	37.9 ± 0.5^b^	15.2 ± 0.2^a^	15.8 ± 0.4^a^	31.1 ± 0.4^a^

Data are presented as mean ± standard deviation (SD) (*n* = 3). Different lowercase letters indicate that TMP had significant differences at different HC times (*P* < 0.05).

## 4. Conclusion

The current study showed that different HC times (0, 5, 10, 15, 20 min; power 550 W, pressure 0.14 MPa) changed the physicochemical properties of TMP, thus affecting the emulsifying properties of TMP emulsions. The results showed that HC changed the structure of TMP, such as pH, particle size, turbidity, solubility, surface hydrophobicity, and reactive SH group. Furthermore, HC increased the EAI significantly (*P* < 0.05) and changed the ESI, droplet size, and rheology of TMP emulsions. Notably, appropriate HC treatment (10 min) decreased significantly particle size and turbidity and increased solubility of TMP (*P* < 0.05), resulting in a significant improvement in the emulsifying properties, according to measurements of ESI, droplet size, and rheology. In addition, HC induced TMP expansion and changed the physicochemical structure of TMP, as confirmed by the research findings of intrinsic fluorescence and CD spectra. This study suggested that HC is a feasible and potential technique, and the research results can provide theoretical basis and technical support for further research on functional modification of TMP and production of emulsified-type meat products.

## Data availability statement

The original contributions presented in this study are included in this article/supplementary material, further inquiries can be directed to the corresponding author.

## Author contributions

YuH: investigation, methodology, data curation, software, and writing—original draft. XR: investigation, resources, and writing—review and editing. YoH, KX, KW, LW, and FW: investigation. FY: conceptualization, methodology, supervision, funding acquisition, and writing—review and editing. All authors contributed to the article and approved the submitted version.
